# Review on Patient-Cooperative Control Strategies for Upper-Limb Rehabilitation Exoskeletons

**DOI:** 10.3389/frobt.2021.745018

**Published:** 2021-12-07

**Authors:** Stefano Dalla Gasperina, Loris Roveda, Alessandra Pedrocchi, Francesco Braghin, Marta Gandolla

**Affiliations:** ^1^ NearLab, Department of Electronics, Information and Bioengineering, Politecnico di Milano, Milan, Italy; ^2^ WE-COBOT Lab, Polo Territoriale di Lecco, Politecnico di Milano, Lecco, Italy; ^3^ Istituto Dalle Molle di studi sull’Intelligenza Artificiale (IDSIA), USI-SUPSI, Lugano, Switzerland; ^4^ Department of Mechanical Engineering, Politecnico di Milano, Milan, Italy

**Keywords:** upper-limb exoskeletons, rehabilitation robotics, neurorehabilitation, robot control, motor recovery, physical human-robot interaction

## Abstract

Technology-supported rehabilitation therapy for neurological patients has gained increasing interest since the last decades. The literature agrees that the goal of robots should be to induce motor plasticity in subjects undergoing rehabilitation treatment by providing the patients with repetitive, intensive, and task-oriented treatment. As a key element, robot controllers should adapt to patients’ status and recovery stage. Thus, the design of effective training modalities and their hardware implementation play a crucial role in robot-assisted rehabilitation and strongly influence the treatment outcome. The objective of this paper is to provide a multi-disciplinary vision of patient-cooperative control strategies for upper-limb rehabilitation exoskeletons to help researchers bridge the gap between human motor control aspects, desired rehabilitation training modalities, and their hardware implementations. To this aim, we propose a three-level classification based on 1) “high-level” training modalities, 2) “low-level” control strategies, and 3) “hardware-level” implementation. Then, we provide examples of literature upper-limb exoskeletons to show how the three levels of implementation have been combined to obtain a given high-level behavior, which is specifically designed to promote motor relearning during the rehabilitation treatment. Finally, we emphasize the need for the development of compliant control strategies, based on the collaboration between the exoskeleton and the wearer, we report the key findings to promote the desired physical human-robot interaction for neurorehabilitation, and we provide insights and suggestions for future works.

## 1 Introduction

When recovering from a traumatic event affecting the ability to perform everyday tasks, the primary goal is to regain functional movements, both at the lower limbs (e.g., walking) and upper limbs (i.e., interacting with daily-life objects). The recovery of motor functionalities is usually possible and relatively straightforward when the traumatic event has an orthopedic source. Still, it becomes trivial when the traumatic event has a neurological basis, for example, after stroke (Cieza et al., 2020). The outcome of the rehabilitation treatment strongly depends on some general neurophysiological aspects of motor relearning. Studies demonstrated that crucial features are high-intensity treatment, repetitive training, involvement and engagement of the patient, and personalization of the therapy according to the user’s residual capability (Langhorne et al., 2009). Given the increasing burden of neurorehabilitation for therapists and the healthcare system, exoskeletons have been proposed since the 90s as a suitable support for post-stroke rehabilitation. Technology-supported therapy aims to provide post-stroke patients with mechatronic devices that help them perform rehabilitation exercises that can potentially foster motor plasticity and improve motor recovery. The efficacy of robot-supported interventions has been widely investigated with randomized clinical trials (RCT) as compared to conventional therapy, and scientific literature reports controversial results (Mehrholz, 2019; Rodgers et al., 2019). Instead, recent systematic reviews and meta-analyses confirmed the suitability of the approach to help patients and therapists during the treatment, showing that the use of robotic devices can positively affect the recovery of arm function in patients with stroke (Bertani et al., 2017; Veerbeek et al., 2017; Wu et al., 2021). A focus on the clinical outcomes of robot-assisted rehabilitation is not the aim of this paper. However, looking at the characteristics of a successful rehabilitation program, if well designed, exoskeletons can provide high-intensity treatment and repetitive training. When coming to the direct involvement of the patient in the control loop (or human-robot interaction strategy) and the personalization of the therapy according to the user’s residual capability, these are important key features, which are still under investigation by the scientific community. Overall, robot-mediated rehabilitation therapy should mimic the quality of conventional therapy performed by physiotherapists and assist patients in regaining lost functions through a wide selection of training modalities. Moreover, it should adapt to patients’ status and recovery stage, both throughout the single movement and over the rehabilitation treatment (Marchal-Crespo and Reinkensmeyer, 2009). In addition, there is a great effort in the scientific community to develop frameworks that take advantage of non-invasive and portable brain monitor techniques (e.g., EEG Noda et al. (2012); Nicolas-Alonso and Gomez-Gil (2012), fNIRS Hong et al. (2020); Khan et al. (2021)). Such approaches are employed to detect user intention (i.e., brain-machine interface) and to directly evaluate motor recovery in terms of neural plasticity, making the framework even more complex. In this work, we will concentrate on upper limbs recovery and assistance, focusing on control solutions for upper-limb exoskeletons—based on physical human-robot interaction—and their hardware implementation.

### 1.1 Upper-Limb Exoskeletons and Human-Robot Interaction

**FIGURE 1:**
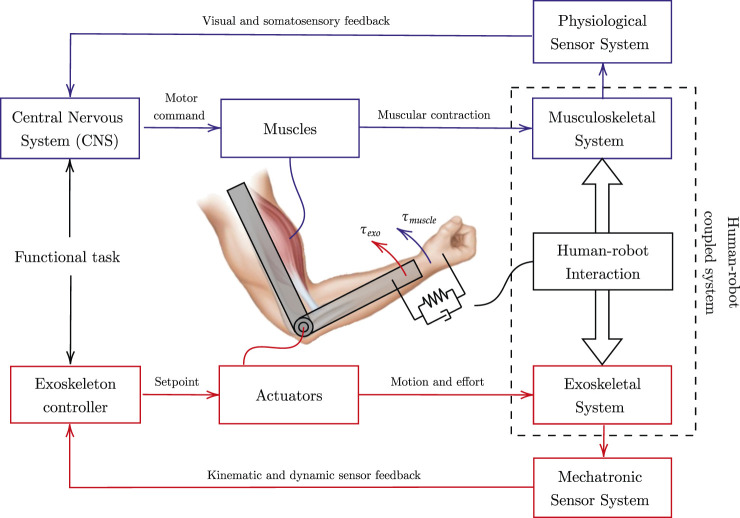
Human-robot interaction representation. The blue scheme represents human motor control, and the red scheme refers to the exoskeleton control. The human-robot coupled system cooperates towards the completion of a shared functional task.

Upper-limb exoskeletons for rehabilitation have been developed to guide patients in accomplishing functional tasks as human-like as possible to foster brain plasticity towards recovery. Exoskeleton solutions that actively guide motion usually consist of serial-connected links that are actuated by powered joints. The exoskeleton and the user are interconnected through one or more interaction ports, generally represented by ergonomic cuffs. At the interaction ports level, the exoskeleton and the user exchange forces and torques. The process by which the human and the robot interact and exchange effort is usually referred to as physical human-robot interaction. First-generation devices were characterized by rigid movements of human segments along a prescribed trajectory, thus resulting in the exoskeleton applying forces/torques at the interaction ports to guide the motion, independently from the effort generated by the user. Thus, one of the critical advancements in robot-assisted research is describing and harmonizing the relationship between voluntary human activity and robot assistance. In fact, robot-assisted movements involved during rehabilitation are characterized by two interactive processes, for which we propose the outline represented in Figure 1. The first process consists of the patient that is encouraged and tries to perform a functional movement, while the latter regards the robot (or the therapist) applying external forces to the patient’s arm to assist and correct the movement (Kahn et al., 2006a).

To complete a functional task, from the human physiological perspective, the intention of the movement is elaborated by the Central Nervous System (CNS), which is in charge of delivering appropriate messages to manage movement execution through its actuating port, and namely the muscles. During movement execution, visual and somatosensory systems provide feedbacks that the CNS analyzes to adjust and correct the strategy according to the comparison between the original intention and the effectively executed movement. The motor control theory is itself an active field of research, and there is discussion whether this comparison is performed accordingly to errors detected at the somatosensory (Gandolla et al., 2014) or kinematic level (Krakauer, 2006), which are two sides of the same coin. Similarly, by mimicking the human motor control scheme, the exoskeleton controller cooperates with the human by superimposing to the muscular effort the (external) robotic contribution, and by shaping the relationship between the human motion and the robot assistance. Regardless of the selected control strategy, the aim is to support the desired motion as revealed by physical human-robot interaction. The control scheme corrects for kinematic or dynamic errors and modulates the set-point signals operated by the mechatronic system’s actuators. During the motion, the muscle-generated torque (*τ*_*muscle*_) interacts with the actuator-generated torque (*τ*_*exo*_), leading to an interactive human-robot coupled system.

### 1.2 Related Works

The rehabilitation process can be divided into three main stages according to time past from the traumatic event, namely acute, sub-acute and chronic phases (Proietti et al., 2016). Generally, the acute phase refers to the first week(s) after the injury. The sub-acute phase includes the range between 15–30 and 180 days after the initial stroke (Péter et al., 2011). The chronic phase is instead defined as the open-ended period starting at about 180 days after initial stroke and characterized by generally slow or no clinical progress (Bernhardt et al., 2017). During these phases, the rehabilitation treatment should make the patient progressively regain the range of motion and muscular strength of the injured limb, and the robot-mediated control strategy should adapt accordingly. In particular, in the earliest stage, since the patient has lost most of the arm functionalities, the robot should help the patient track a predefined trajectory to improve the limb range of motion and reduce muscular atrophy or tendon retractions. Recent studies demonstrated that patients undergoing early robot-mediated therapy within the first weeks after the trauma could gain greater reductions in motor impairment and improvements in functional recovery of the upper-limb (Masiero et al., 2007). As soon as the patient has regained some voluntary muscular contractions, but the generated strength is not adequate to perform precise and complete movements and consequently not sufficient to fulfill functional tasks, the robot should provide the assistance needed to complete the movement, as a physical therapist would do. Moreover, to engage the patient and better induce neural plasticity, the robot should encourage the users to initiate the movements with their active muscular efforts and progressively provide decreasing assistance until the patient has regained the lost functionalities. In fact, it has been demonstrated that the carryover effect is selectively obtained when the patient program the movement and perceives the external assistance as a part of their control loop (Gandolla et al., 2016b). Finally, when stroke survivors have regained most of the range of motion they could recover, the robot should help them recover muscle strength. Recent works demonstrated that improvement in motor function was possible even at late chronic stages, i.e., after the 3–6 months critical window (Ballester et al., 2019; Gandolla et al., 2021). In this situation, the patient actively performs the exercises against resistive forces provided by the robot. Further, challenging strategies can be used to involve and engage the users to continue the rehabilitation treatment. There also exists a branch of robot-assisted rehabilitation that involves other therapeutic approaches combined with upper-limb exoskeletons. For example, Functional Electrical Stimulation (FES) has been used to enhance functional recovery of the paretic arm in stroke survivors (Howlett et al., 2015). The action of FES, combined with the residual voluntary effort of the user, has proven to enhance cortical plasticity (Gandolla et al., 2016b). For example, Ambrosini et al. (2021) demonstrated that EMG-triggered FES combined with anti-gravity robotic assistance could improve the therapeutic effects post-stroke rehabilitation. However, these approaches involve a third interactive process, i.e., the FES-induced muscular contraction, that must be integrated with the robot controller and the user’s voluntary actions. For this reason, we will not include in detail FES-based robot-mediated rehabilitation in this work. Overall, it is clear that the design of effective training modalities plays a crucial role in robot-assisted rehabilitation and strongly influences the treatment outcome.

While several reviews on upper-limb exoskeletons are available, most of them deal with the mechanical design of the robotic systems Lo and Xie (2012); Van Delden et al. (2012); Brackenridge et al. (2016); Iandolo et al. (2019); Gull et al. (2020) or with their efficacy in clinical practice Maciejasz et al. (2014); Rehmat et al. (2018). Other reviews investigate robot-mediated rehabilitation control strategies, but they propose taxonomies and classification that are not consistent, and they typically present control methods at high-level of implementation Marchal-Crespo and Reinkensmeyer (2009); Basteris et al. (2014); Proietti et al. (2016); Miao et al. (2018). In particular, with “high-level” strategies, the literature usually refers to those control methods that shape the human-robot interaction behavior and focus on specific training modalities.

For instance, Marchal-Crespo and colleagues presented a review on robotic training strategies Marchal-Crespo and Reinkensmeyer (2009). The authors specifically target the review to “high-level” strategies, i.e., such “aspects of the control algorithm that are explicitly designed to provoke motor plasticity”. Their work mainly focuses on assistive controllers classified as 1) impedance-based, 2) counterbalance, and 3) EMG-based methods. According to the authors, the impedance-based controllers create restoring forces when the participant deviates from the desired exercise trajectory, but they do not intervene if the subject is moving along the desired path. Counterbalancing controllers, instead, provide weight compensation to the upper-limb through passive elastic elements or active control schemes, but they do not help the participant follow the task trajectory. Finally, EMG-based controllers involve surface electromyography signals (sEMG), and they are aimed at enhancing the residual muscular torques of the participant.

In a different recent systematic review, Basteris et al. (2014) focused on training modalities in robot-mediated upper-limb rehabilitation and they proposed a classification framework based on the expected subject’s status during human-robot interaction. In their work, training modalities are divided in four macro categories: 1) active, 2) active-assistive, 3) passive, and 3) resistive. In active mode, the robot does not apply force to the subject’s limb and behaves compliantly with the user’s movements. In active-assistive mode, it provides assistance towards the completion of the task. In contrast, in passive mode, the robot performs the movement without accounting for the subject’s activity, while in resistive mode, it provides forces opposed to the movement. The authors also underline that the literature lacks information regarding the implementation of the different modalities by different research groups.

Another example of review regarding upper-limb exoskeleton control strategies has been proposed by Proietti et al. (2016). The authors presented a taxonomy based on three main global rehabilitation features: 1) assistance, 2) correction, and 3) resistance. While assistance refers to the ability of the robot to support the weight of the limb and provide forces to complete the task, with correction strategies, the robot does not assist the patient, but it corrects the movement to follow a desired path and to provide coordination among joints. Finally, resistance concerns the robot acting against the desired movement. However, the authors state that such features are often combined to properly render the desired human-robot interaction.

### 1.3 Aim of the Review

At this stage, it is clear that different research groups presented different taxonomies and classifications, which are not consistent among works. One of the most challenging aspects of reviewing control strategies for rehabilitation exoskeletons is to provide the understanding of the control method, which most of the time is embodied in nested control algorithms and strongly depends on the available robot hardware. In fact, none of the reviews presented in the literature spans from “high-level” training modalities, to “low-level” control scheme implementation, to “hardware-level” implementation, to the characterization of the needed sensor systems, and they do not provide a match between these aspects. The objective of this review is to provide a multi-disciplinary vision of patient-cooperative control algorithms for upper-limb rehabilitation exoskeletons. The aim is to bridge the gap between human motor control aspects, rehabilitation training modalities, and robot development. To this aim, we propose a three-level classification (Table 1). The first level deals with literature high-level human-robot interaction training modalities, which directly relate to the desired behavior of the rehabilitation exercise and to the capability of the robotic exoskeleton to induce motor recovery according to the patients’ status. Such high-level modalities are in turn embodied by low-level control strategies, which promote a large variety of physical human-robot interaction according to the residual capabilities of the user. Thus, in the second level, we focus on low-level control schemes that are exploited to promote compliant motion and to display the desired human-robot behavior. Instead, in the third level, namely hardware-level, we draw some insights regarding the state-of-the-art hardware implementation, mainly focusing on actuation, transmission and sensor system technologies. Finally, we outline how different research groups could achieve the desired physical human-robot interaction with their developed hardware. To this aim, we review some upper-limb exoskeleton works as examples of possible different choices made at the three proposed levels. Indeed, to promote the desired human-robot interaction behavior, different approaches can be followed at different levels of implementation.

**TABLE 1:** Presented classification of control methods for patient-cooperative compliant robotics for upper-limb rehabilitation.

Term	Description
”High-level” training modalities	Control strategy that does not necessarily depend on the developed hardware. Directly relates to the desired human-robot interaction behavior during the rehabilitation exercise. Explicitly designed to induce motor plasticity according to the stage of the recover process, and to improve the treatment outcome
”Low-level” control strategies	Control strategy that depends on the developed hardware. Baseline control law that represents a substrate for implementing a variety of ”high-level” modalities. Relates to the capability to promote shared, cooperative, compliant motion between the subject and the robot
”Hardware-level” implementation	Hardware implementation and control approaches used to promote transparency and compliant motion. Relates to actuation, transmission and sensor technologies involved in the development of compliant joints for rehabilitation exoskeletons

## 2 High-Level Rehabilitation Training Modalities

High-level training modalities have been proposed to promote motor recovery at different stages of the rehabilitation treatment, taking inspiration from neuroplasticity and neurophysiological aspects that are explicitly involved during motor relearning after stroke (Krakauer, 2006; Reinkensmeyer et al., 2016). What researchers want to achieve is to maximize the outcome of the rehabilitation by actively involving the patient in the process and by minimizing the robot effort needed for the completion of the rehabilitation task. To cope with this objective, the robots should cooperate with the subjects during the treatment as a therapist would do. High-level training modalities are usually classified according to the physical interaction between the subject and the robot during the rehabilitation training. Thus, most researchers relate rehabilitation modalities to the subject’s status and engagement (Marchal-Crespo and Reinkensmeyer, 2009; Basteris et al., 2014; Trigili et al., 2019), others to the robot’s behavior (Pirondini et al., 2016). However, each research group presents a different classification, which leads to non-coherent and misaligned literature taxonomy. In this review, we posit that upper-limb exoskeletons for rehabilitation can mainly operate in four macro-modalities: 1) passive, 2) active-assistive, 3) active, and 4) resistive, according to the human-robot interaction behavior, which are summarized in Table 2.

**TABLE 2:** High-level training modalities for upper-limb robot-mediated rehabilitation. Classification refers to subject’s status at interaction. Red arrows represent exoskeleton assistance (solid) or resistance (dashed). Blue arrows indicate user voluntary effort, if present.

High-level modalities	Passive	Active-assistive	Active	Resistive
**Features**	The robot performs the task without accounting for subject’s effort. The robot corrects trajectory errors	The robot and the subject perform the task cooperatively. The robot can provide weight counterbalance or trajectory-based corrective assistance	The subject actively performs the task. The robot does not provide assistance nor resistance to the subject. No time-dependent trajectory is present	The subject actively performs the task. The robot resists to the movement by providing opposing forces
**Human-robot interaction**	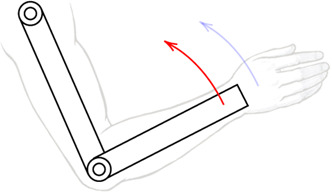	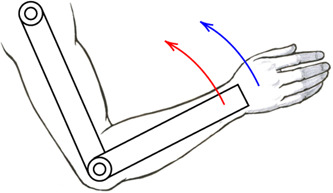	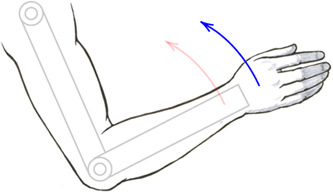	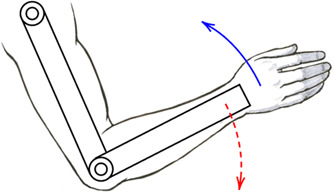
**Rationale**	Prevents soft tissue stiffening. Passive mobilization generates somatosensory stimulation	Preserves subject motivation and self-esteem. Subject’s involvement promotes motor learning	The robot is a measurement device. Permits ROM exploration and do not limit subject’s voluntary free movements	Promotes subject’s involvement and potentiate muscular strength
**Variants**	Passive-triggered strategies, Teach-and-replay strategies, Passive-mirrored strategies	Purely corrective and weight counterbalance assistance, Inter-joint coordination assistance, Adaptive assistance	Tunneling or trajectory-constrained strategies	Viscous-field and error-augmentation strategies

### 2.1 Passive Modalities

One of the first approaches used in neurorehabilitation regards passive mobilization of the patient’s limb along a desired trajectory (Lum et al., 2006). The term “passive” refers to the subject’s interaction status, by which the exoskeleton is “active” and performs the movement without accounting for the subject’s intention of action. The robot provides stiff behavior and applies high corrective forces to follow the desired trajectory (Marchal-Crespo and Reinkensmeyer, 2009). However, passive mobilization has been proven to limit one of the most important mechanisms of motor relearning: it prevents participants to program in advance the movement, thus it limits the capability to learn from their mistakes, which are driving signals for motor learning (Shadmehr et al., 2010). In fact, the CNS creates an internal model of the environmental dynamics and, during human motor adaptation, it learns to anticipate the movement according to somatosensory and kinematic errors. (Patton et al., 2006; Emken et al., 2007b). In a clinical setting, passive mobilization is usually only operated during the first stages of motor recovery. The rationale of early mobilization is that passive stretching of the limb can prevent stiffening of soft tissue and it helps to reduce spasticity and tendon retractions (Masiero et al., 2007). Moreover, repetitive movements of the limb can generate somatosensory stimulation that can potentially induce brain plasticity and help patients re-learn the desired muscular activation patterns (Bastian, 2008; Crespo and Reinkensmeyer, 2008). Different variants of passive mode are present in literature.

#### 2.1.1 Passive-Triggered Mode

The passive-triggered mode consists in the wearer that triggers the exoskeleton assistance as in passive mode (Proietti et al., 2016). This encourages the participant to self-initiate movements, which is an essential feature for motor relearning (Marchal-Crespo and Reinkensmeyer, 2009). The trigger can derive from both cognitive or physical human-machine interfaces. On the one hand, participants can initiate the movement by means of movement intention detection that can be performed by means of gaze-tracking systems (Frisoli et al., 2012; Novak and Riener, 2013), Motor Imagery based Brain Computer Interface (MI-BCI) (Barsotti et al., 2015; Brauchle et al., 2015), or tongue-based interfaces (Ostadabbas et al., 2016). Alternatively, the passive assistance can be triggered by allowing the participants to attempt a movement with their residual muscular force (i.e., without any robotic support) and initiate the movement after some performance conditions are met. In particular, the movement can be triggered by spatial trajectory tracking errors (Kahn et al., 2006b), movement speed (Krebs et al., 2003), residual forces of the participant (Colombo et al., 2005; Chang et al., 2007) or EMG-based intention detection (Dipietro et al., 2005; Gandolla et al., 2016a). We underline that the triggered assistance is generally applied to passive mobilization of the arm, but it can be also applied to controllers that apply different levels of assistance and resistance to support the arm motion, such as active-assistive controllers.

#### 2.1.2 Teach-and-Replay Mode

Different methods exist to define the reference trajectories to be followed by the robot in passive mode. In teach-and-replay mode, joint trajectories are created by recording the robot joint angles during a teaching phase. In this phase, the robot is generally operated in transparent mode (the controller compensates for the robot weight and dynamics) not to resist external forces and to undergo external motion. The therapist guides the affected arm in the workspace, and the desired trajectory is recorded from the exoskeleton joints. In some approaches, relevant way-points are determined, and the trajectory is optimized through a minimum-jerk algorithm to avoid undesired oscillations and achieve natural human-like movements (Nef et al., 2007; Lin et al., 2021). Then, the robot actively performs the task taught by the therapist, replays the joint trajectories, and corrects trajectory deviations with corrective gains (Kumar et al., 2019). The therapist can usually tune the execution velocity of the task to match the patient’s needs (Nef et al., 2007; Xu et al., 2011). When the desired movement is registered apriori by the contralateral arm (i.e., the healthy one), this modality can also be addressed as record-and-replay mode (Proietti et al., 2016).

#### 2.1.3 Passive-Mirrored Mode

A different option is the passive-mirrored mode, which can be implemented only with exoskeletons provided with two arms (Van Delden et al., 2012). The strategy consists of passively mimicking the behavior of the healthy limb by supporting the impaired one passively (Proietti et al., 2016). Usually, this mode can also be referred to as “master-slave” mode since the desired trajectory is continuously computed and commanded by its contralateral side, which is generally operated to behave transparently to the healthy limb (Colizzi et al., 2009; Kumar et al., 2019).

### 2.2 Active-Assistive Modalities

**FIGURE 2:**
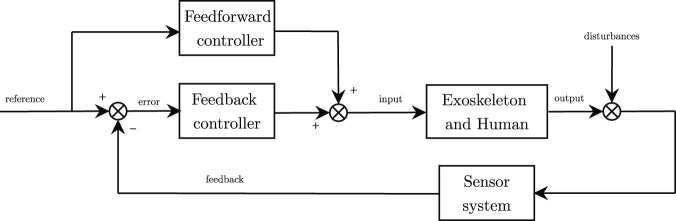
General control scheme. Feedback control (impedance-based corrective assistance) and feedforward control (counterbalance assistance) sum up to compute the desired control input.

Since passive controllers do not involve active participation from the patient, the literature suggests that more complex control strategies based on subject’s involvement could lead to better results, at least after the first stages of the rehabilitation process (Huang and Krakauer, 2009; Reinkensmeyer et al., 2016). This is the case of assistive controllers, by which participants are involved in the completion of the task, while the robot partially assist them in the completion of the task. Due to their nature, assistive strategies guarantee compliant interaction between the human and the robot, and they permit deviation from the desired trajectory (if it exists). As previously mentioned, this feature is a key concept for motor learning as it preserves patients motivation and self-esteem while forcing them to actively adapt their internal models to minimize kinematic tracking errors (Krakauer, 2006; Shadmehr et al., 2010). Similarly to what introduced by Marchal-Crespo and Reinkensmeyer (2009), we further divide active-assistive modalities in two different groups: 1) weight counterbalance assistance, which introduces an offset compensation that counterbalances the weight of the arm; 2) trajectory-based corrective assistance, which generates a force-field environment that helps the user follow the desired trajectory, and 3) inter-joints coordination assistance, which regulates the coordination of the joints and promotes physiological synergies during trajectory-based and free movements. From a more control-based perspective, active-assistive modalities can thus be implemented through a feedback loop combined with a feedforward contribution, as shown in Figure 2. The feedback closed-loop regulates the position or the interaction forces along the reference exercise trajectory (i.e., impedance-based correction), while the feedforward loop compensates for perturbation with a model-based prediction, such as weight counterbalance assistance and friction compensation.

On top of this general control scheme, several additional features can be added to achieve inter-joint coordination, to implement mirror-based or teach-and-play strategies, or to adapt the assistance according to the treatment outcome. Most exoskeleton prototypes can be operated by a combination of these features.

#### 2.2.1 Weight Counterbalance Assistance

In the first case the robot provides the effort only to compensate for weak muscular tone that is unable to support the weight of the arm. In purely counterbalancing strategies, there is no trajectory tracking correction, and the user can actively explore the range of motion. Weight counterbalance is usually implemented through feedforward compensation of the arm weight and dynamics. Several anti-gravity compensation algorithms are available in literature. Most methods are based on dynamic models of the robot-patient system (Just et al., 2017). To compensate for the robot dynamics, its mechanical properties (masses, centers of mass and inertia tensors of each joint-link) are usually extracted from the CAD model of the robotic system (Nef et al., 2007; Just et al., 2016), while weights and lengths of the human arm can be derived from literature anthropometric tables, such as (Winter, 2009). Once the dynamic properties of the two interacting systems are obtained, they are fed in to geometric (Moubarak et al., 2010), Lagrangian (Nef et al., 2007) or recursive (Kim and Deshpande, 2017) inverse-dynamics algorithms to compute the desired joint torques to compensate for the gravity of the human-robot system. However, mathematical models do not always entail a real experience of weight relief for the end-user, and methods to compensate for inertia and load uncertainties have been developed for safe and accurate control of upper-limb exoskeletons. For instance, Wang and Barry (2021) developed a *H*_*∞*_ robust adaptive controller that can adapt to the inertia and load uncertainties and compensate for their effects. In a simulation study, the authors proved that such adaptive controllers could be applied to safe and reliable motion control of rehabilitation exoskeletons. Other approaches are instead based on measurements from force-interfaces (Ragonesi et al., 2013; Just et al., 2020), and combine the experimental data to accurately identify the gravity term without extracting mass and inertia parameters (Moubarak et al., 2010).

Yet another solution to the weight balancing problem is using passive elastic elements to generate additional torques to counterbalance gravity. For instance, the RETRAINER (Ambrosini et al., 2017; Puchinger et al., 2018) employs passive springs to compensate for the wearer weight. Other examples, such as the Pneu-WREX (Sanchez et al., 2005; Wolbrecht et al., 2008) or the BLUE SABINO exoskeletons (Perry et al., 2018; Hill et al., 2019), employ elastic elements in combination with active controllers.

#### 2.2.2 Trajectory-Based Corrective Assistance

In trajectory-based corrective strategies, the user has to follow a desired trajectory and the robot corrects undesired behavior, similarly to a position control system, but with a more compliant behavior (Marchal-Crespo and Reinkensmeyer, 2009). The exoskeleton usually does not intervene as long as the patient is following the correct movement. In fact, to accommodate human variability in performing movement, a deadband is usually introduced where the user can move freely. Outside the deadband, if the subject deviates from the target trajectory, the system produces a gradient of restoring forces that usually vary proportional to the trajectory deviation (Nef et al., 2007). Despite the low-level control strategy, the robot is usually commanded to recover from kinematic errors through a virtual zeroth order impedance (i.e., a spring), namely implementing pure stiffness control. The controller implements a corrective action or force-field to guide the user along a desired trajectory or path. By relaxing the corrective gains of the exoskeleton (i.e., by lowering the virtual stiffness), the system displays a more compliant behavior. More recent upper-limb exoskeletons include also corrective controllers provided with viscous force-fields that dampen and stabilize the movements (Proietti et al., 2015; Kim and Deshpande, 2017).

#### 2.2.3 Inter-joint Coordination Assistance

Jarrassé et al. (2014) presented a review of studies on upper-limb coordination in stroke patients, intending to illustrate the potential of robotic exoskeletons to rehabilitate inter-joint coordination. Usually, inter-joint coordination can be addressed as a kinematic problem that promotes the activation of physiological muscular synergies compromised by the stroke event. However, most training strategies focus on supporting all the joints of the exoskeletons independently. Very few approaches have attempted to address the spatio-temporal relationship between joints, and the clinical efficacy of this approach is still questionable (Jarrassé et al., 2014). Since most active-assistive controllers follow a reference trajectory, one of the simplest ways to promote inter-joint coordination is to generate a set of joint trajectories that respect specific coordination and time-dependency among them. However, computing such joint trajectories is a significant issue. They can be recorded from physiological movements performed by healthy subjects, or the therapist can guide them in a teach-and-replay fashion, or they can be computed through optimal inter-joint coordination inverse-kinematics planners. However, these approaches still require programming specific movements in advance and need to be re-computed for each task or exercise. Consequently, they limit the patients’ freedom of movement with the exoskeleton, and they do not investigate the inter-joint coordination problem as a whole.

For instance, Brokaw et al. (2013) developed a Time Independent Functional Training (TIFT) method that provides focused training of inter-joint coordination after stroke and permits movement only if a good level of coordination is achieved. In detail, TIFT provides joint-space walls that resist movement patterns that are inconsistent with the targeted shoulder-elbow inter-joint coordination pattern. Time independence is added to promote voluntary motion from the user without constraining the patient’s arm to a fixed, rigid trajectory. Similarly, Crocher et al. (2010) proposed a controller which allows to impose velocity-based coordination through viscous force-field without constraining end-point motion. Specifically, the controller does not impose any trajectory, but it reacts user-applied forces by generating joint torques that restrict the motion when a certain velocity-based inter-joint coordination is not obtained. The same approach was used to perturb the human natural inter-joint coordination in healthy subjects Proietti et al. (2017). Results showed that the controller did not directly constrain end-effector movements, but it applied inter-joint velocity-dependent perturbing force fields distributed at the joint-level that disturbed the users’ natural upper-limb coordination strategy.

Instead, besides the existence or not of a pre-defined desired trajectory, Kim and Deshpande (2015) presented a control strategy for the shoulder mechanism of an upper-body exoskeleton to assist in achieving coordinated motion at the shoulder complex. The idea is to introduce a coupling torque according to an impedance-based control law that adjusts the shoulder scapulohumeral rhythm configuration. The reference position for the shoulder elevation is computed according to an experimentally obtained quadratic law that correlates the shoulder elevation angle to the humerothoracic arm elevation. Such a relationship can be included and actuated both during free-space motion and along with exercise trajectories. In the first case, the controller implements a reactive action that corrects undesired postures with inter-joint coordination torques (Kim and Deshpande, 2017). The user can explore the range of motion using all the exoskeleton joints, and the corrective torques are applied only at certain joints to maintain the desired coupling. In the latter case, a proper inverse-kinematics algorithm includes inter-joint coordination constraints within the optimization problem. The algorithm exploits the kinematic redundancy of the robot (e.g., through the swivel angle) to reconfigure the exoskeleton according to the scapulohumeral rhythm and computes the desired joint trajectories (Dalla Gasperina et al., 2020).

#### 2.2.4 Assistance Adaptation

However, to optimize the outcome of motor learning and to avoid the “slacking” effect, the assistance should be tailored to each stroke patient throughout the movements and over the rehabilitation treatment. Namely, the slacking behavior of the human motor control regards the patient that, trying to optimize the effort to accomplish a task, may learn to provide only the strictly sufficient amount of force needed to complete the task and it takes advantage of the exoskeleton assistance, which performs most of the physical effort (Marchal-Crespo and Reinkensmeyer, 2009). To avoid such a phenomena, the assistance should be supplied only when the subject is not able to actively complete the task and tailored to recovery stage. Different approaches for assistance adaptation have been explored in literature. They mainly involve trial-by-trial adaptation to modulate the robot assistance according to some user-specific performance metrics. For example, adapting control parameters is a key aspect of patient-cooperative strategies, by which the assistance can be automatically tailored to the participant’s performances and needs. The goal is to keep the users engaged and actively participating to the treatment, by providing the minimum assistance level to fulfill the task and, at the same time, by promoting the maximum achievable patient muscular effort (Proietti et al., 2016). Adaptive assistance strategies are also referenced as assisted-as-needed strategies and are usually governed trial-by-trial through the following general adaptation control law:

$$u_{i}=fu_{i-1}+gE_{i}$$ (1)

where *u*_*i*_ is the assistance (or control parameter) that is adapted over time, *E*_*i*_ is a performance error or metric that can denote the capability of the participant to initiate the movement, to follow a desired path, or to reach a target, and *i* indicates the *i* ^*th*^ trial. *f* is a forgetting factor (0 < *f* < 1), included to avoid slacking and to promote continuous involvement of the participant and *g* is the gain that determines the reaction timing of the adaptation control law. Including the forgetting term is a key feature to challenge the participant, even if performance errors are low. Indeed, if we consider removing the forgetting term (i.e., *f* = 1), the control parameters can saturate to the configuration that optimizes the performances, without taking into account the participant effort and engagement. According to the previously described taxonomy, adaptation can occur at both feedback and feedforward assistance loops. In the first case, robot stiffness and corrective force-fields are tuned according to the participants’ abilities and effort. For instance, Krebs et al. (2003) first proposed a performance-based control algorithm, which tunes the corrective assistance according to speed, time, or EMG signals. Similarly, the correction can be tuned trial-by-trial according to error-based kinematic performance metrics (Proietti et al., 2015; Pérez-Ibarra et al., 2019). For example, the adaptation control law can rely on terms related to the difference between the measured trajectory and the one desired to fulfill the task, the normalized distance from a specific target, or indexes that indicate the accuracy in drawing a geometric shape (Stroppa et al., 2017b, 2018).

Alternatively, the adaptation can be applied to the feedforward assistance, as presented by Wolbrecht et al. (2008). The authors implemented an *assist-as-needed* controller that adapts the feedforward assistance, which is computed using radial basis functions and learned on subject’s abilities. They added an error-based learning factor, which iteratively adapts the feedforward contribution, and a force decay, which reduces the support when the subject is able to perform the movement correctly.

### 2.3 Active Modalities

Active modalities, also known as “transparent” modalities, are characterized by a “human-active/robot-passive” behavior. The robot does not provide assistance, nor resistance to the movement, and the subject is allowed to perform movements without perceiving the robot effort. Active modalities can be beneficial as they enable the exoskeleton to become a measurement device (Nordin et al., 2014). Recent studies demonstrated that kinematic data can bring meaningful information to clinical assessment in post-stroke rehabilitation (Bigoni et al., 2016). De Oliveira et al. (2021) demonstrated that exoskeleton joint angle data are accurate measurements of arm and shoulder kinematics. However, when the robots are operated in active mode for assessment purposes, transparency is a fundamental feature. If the robot provides non-zero torque biases while the wearer is being evaluated, it generates undesired resistances during the upper-limb motion of subjects and it can consequently influence the performance and consequently the assessment (Proietti et al., 2016). When the robot is operated in active mode, the range of motion of each joint can be tuned and limited by control to avoid that the wearer overcomes physiological limits. Usually, range of motion boundaries are implemented through virtual walls, which can be implemented through repulsive virtual spring-damper systems.

#### 2.3.1 Tunneling Strategies

As we previously described, corrective strategies usually provide assistance to help the subject follow the desired trajectory both along longitudinal and orthogonal directions. Conversely, the so-called tunneling strategies usually permit free movements, and they provide correction only when boundaries conditions are met. The concept is to create a virtual cylindrical channel at the end-effector that permits free active movements along the longitudinal direction, but restricts movements in radial directions by applying restoring forces to the end-effector position, if the user exits the virtual channel. In order words, tunneling strategies permit active free movement and bound the task-space or joint-space workspace with software virtual walls and boundaries. Since these strategies do not assist the movement along the trajectory main direction, as stated by Proietti et al. (2016), the concept is linked to time-independence of the task references. In particular, in such modalities, there are not trajectory profile references that relate position, velocity, and time. Instead, the controller is fed with a time-independent three-dimensional desired path. Guidali et al. (2011) implemented a tunneling strategy by subdividing the task in multiple sub-movements, and creating force-fields channels to correct the hand position within each sub-movement. Then, after the user had reached a way-point, a trajectory generator algorithm updates the trajectory for the next sub-movement. Similarly, Wu et al. (2018a) implemented a three-dimensional channel based on three concentric channels. The inner channel permits active free movements, the central one assists the user to reach the inner channel, while the outer channel restricts movement directed out of the virtual tunnel. In some works, a timeout-triggered assistance, also known as *back-wall*, is added to help the users to complete the task when they get stuck and they are not able to actively initiate or finish the movement. The *back-wall* is usually implemented through a pushing force along the longitudinal direction of the channel (Proietti et al., 2016). If such timeout-triggered assistance is present, tunneling strategies can be become assistive as well. Thus, the taxonomy can be confusing and it can be difficult to distinguish purely tunneling strategies, with or without *back-wall*, from active-assistive modalities.

### 2.4 Resistive and Challenging Modalities

Historically, rehabilitation robots were designed to assist the patient during the initial phases after stroke, i.e., when the patient is severely impaired and needs substantial assistance to complete functional tasks. Then, when the patient has (hopefully) relearned most of the lost motor functionalities but still has to gain some muscular tone, conventional therapy proposes gym-like body-weight exercises. Resistive modalities have been recently introduced as a rehabilitation solution for the latest stages of the motor recovery process to engage the patients during their progression through robot-mediated exercises. In fact, robots can provide an aquatic therapy-like environment that allows user-driven free movements with or without viscous resistance (Kyoungchul et al., 2010). Usually, resistive modalities do not follow trajectory references. Still, they permit the user to actively explore the workspace, and the exoskeleton resists user’s movement through virtual viscous force-fields, which are usually inversely proportional to the movement velocity (Song et al., 2014). Finally, we could include in this category also other challenging strategies based on error-augmentation methods since they indirectly resist the motion by repressing the voluntary movement or by emphasizing kinematics errors. Error-augmentation consists of algorithms that, through repulsive forces, amplify movement errors rather than decrease them (Patton et al., 2006). Indeed, as previously mentioned, motor learning has underlined that kinematic errors are fundamental neural signals to improve motor adaptation (Emken et al., 2007a). A similar approach involves instead the implementation of task-space force fields that push the user’s arm away from equilibrium points or comfortable positions to enhance workspace exploration (Wright et al., 2015, 2018). Resistive and challenging modalities have been broadly investigated in gait and locomotion analysis. However, few studies have been performed in upper-limb functional rehabilitation (Abdollahi et al., 2014; Israely and Carmeli, 2016).

## 3 Low-Level Robot-Assisted Control Strategies

To guarantee a good collaboration of subject and exoskeleton during physical human-robot interaction, the robot should display a wide range of haptic mechanical impedance, which should span from high-compliance (low-resistance) to high-stiffness behaviors. While achieving rigid control can be considered trivial, promoting the so-called compliant motion, i.e., the robotic device should behave transparently to voluntary human activity, can be challenging. Furthermore, its performances are strongly related to the mechanical design of the actuation unit and thus they depend on the specific hardware implementation. Namely, compliant control refers to the capability of the robotic system to generate movement and, simultaneously, to undergo movement if external forces are applied. On the one side, the robot drives the motion of the limb and corrects for trajectory errors. On the other side, the user applies forces/torques to the robot, which should permit deviations from a defined equilibrium point without suppressing the voluntary activity. Since compliant motion doesn’t limit in any way any intention of movement of the interacting user, it guarantees one of the most fundamental features for efficient motor recovery and demonstrated to be a fundamental, yet challenging, feature in rehabilitation robotics. To make the processes mentioned above interact smoothly, each of them should be aware of the other’s behavior. While the human, thanks to its somatosensory and visual systems, can directly feel and monitor the behavior of the robot, both in terms of interaction forces and perceived movements, the robotic device needs an adequate sensors network to detect the involvement and the intention of movement coming from the user. Indeed, exoskeleton developers can follow different approaches to detect the user’s intention of movement, which deeply depend on the implemented hardware.

Recently, Calanca et al. (2016) published a survey that presented the state of the art of compliant control algorithms according to the available sensor networks and control schemes. The authors analyzed solutions from traditional robotics, usually involving stiff joints, to more recent approaches that combine soft joints with advanced control schemes. Indeed, the concept of compliant motion refers to the capability of a system to shape the dynamical relation between motion and torque/forces, instead of independently controlling the joint motion or the joint torques of the robot. Thus, to promote compliant interaction between the human and the robot, along trajectories or in free motion, several low-level control strategies have been proposed (Miao et al., 2018). Most of the compliant controllers, instead of relying on high-gains corrective position control, implement nested control loops that are usually characterized by an inner high-accuracy loop, which guarantees fast response of the robotic system, and an outer “flexible” loop, which includes the human contribution and implements the interaction control. Such approaches mainly rely on two control schemes: Impedance control (force/torque based control) and its dual admittance control (position based control) Ott et al. (2010); Schumacher et al. (2019). However, as we previously introduced, the perceived compliance can be implemented either through compliant controllers, or through mechanical compliance, for example by using soft joints instead of stiff joints (Calanca et al., 2016, 2017; Schumacher et al., 2019). Thus, in this review, we include and discuss position control of soft joints as it can itself promote compliant interaction control.

### 3.1 Impedance Control

Among all, impedance control is one of the most common approaches, and it has been demonstrated to be a very efficient solution for neurorehabilitation (Marchal-Crespo and Reinkensmeyer, 2009; Mehdi and Boubaker, 2012). It implements dynamic control that shapes the desired mechanical impedance through human-robot interaction: a torque/force output is generated from a position input. In this section, we will firstly describe the main features of impedance control applied to a joint of the robot (i.e., in the joint-space), then we will explain its applicability in the Cartesian-space.

**FIGURE 3:**
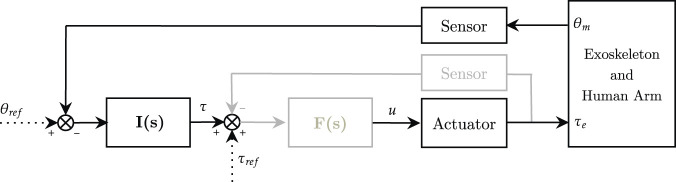
Impedance control scheme in the joint-space. Implicit (black) and explicit (black and gray). **I**(*s*) is the impedance controller, **F**(*s*) is the force controller (only explicit). *θ*_*ref*_ and *τ*_*ref*_ represent respectively reference angular position and torque, while *u* refers to the motor corrent control signal.

Impedance control was first introduced by Hogan (1985), and it is also referred to as force-based position control or equilibrium point control. Indeed, differently from traditional position control, this approach does not aim at precisely tracking trajectories, but it proposes a trade-off between interaction forces and deviation from the reference motion. To promote this behavior, impedance control is characterized by a nested loop architecture. An inner torque-feedback loop implements the transparent behavior and promotes the mechanical compliance (i.e., it “softens” the control). An outer position-feedback loop corrects for trajectory tracking errors by applying forces or torques aimed at the completion of the task (i.e., it “stiffens” the control). Two different variants of the impedance control can be identified. When the actuation unit is inherently back-drivable, the torque control can be implemented through an open-loop control loop (i.e., implicit impedance). In the other cases, a loadcell or an elastic element is exploited in series as a feedback signal for the closed-loop torque control loop (i.e., explicit impedance) (Khalil and Dombre, 2002). Explicit impedance control improves force sensitivity, but can jeopardize the coupled stability of the human-robot system. In fact, high torque-loop control gains can cause stability issues when in contact with hard surfaces (Calanca et al., 2016; Focchi et al., 2016), thus there exists a trade-off between torque fidelity tracking and stability of the impedance controller. The impedance control schemes (implicit and explicit) can be implemented in the joint-space as shown in Figure 3.

The reference or equilibrium joint position is *θ*_*ref*_, while the actual position *θ*_*m*_ is usually measured by motor encoders. The torque control signal *τ* is usually computed as:

$$\tau =I\left(s\right)\left(\theta_{ref}-\theta_{m}\right)+\tau_{ref}$$ (2)

where *I*(*s*) is the mechanical impedance model, usually multiplied by the trajectory tracking error, and *τ*_*ref*_ represents the torque reference, often used to compensate for gravity and friction torques. The actuator block represents the actuator dynamics and converts the control signal *u* to the desired output. If the explicit impedance control scheme is exploited, *F*(*s*) represents the inner torque control loop, which is in charge of making sure that the measured torque output (*τ*_*e*_) tracks its reference (*τ* + *τ*_*ref*_). The *F*(*s*) estimates the target torque of the actuator (*u*), usually through a Proportional–Integrative–Derivative (PID) controller. The impedance filter *I*(*s*) is generally described by a *n* ^*th*^ polynomial system that varies according to the order of the virtualized mechanical impedance system. Impedance control of 0^*th*^ order, also referenced as pure stiffness control (Trigili et al., 2019), is formally equivalent to a proportional (P) position controller, where the gain represents the desired mechanical stiffness *K*_*s*_ (Eq. 3).

$$I\left(s\right)=K_{s}$$ (3)

If a 1^*st*^ order impedance is implemented, the velocity error, namely ${\dot{\theta }}_{ref}-{\dot{\theta }}_{m}$ , is multiplied by a target damping coefficient *K*_*d*_, which is usually aimed at reducing jerky oscillations and dissipating spring energy. The 1^*st*^ order impedance control formally corresponds to a proportional-derivative (PD) position controller (Eq. 4).

$$I\left(s\right)=K_{s}+sK_{d}$$ (4)

This is one of the most common implementations in rehabilitation robotics as the virtual stiffness, by means of the virtual spring constant *K*_*s*_, pulls the joint link towards its reference (i.e., the spring corrects for deviations from the joint trajectory), while the virtual damper *K* − *d* dampens oscillations and stabilizes the movement. However, in most cases, since the desired velocity ${\dot{\theta }}_{ref}$ is not accessible, the desired velocity can be neglected and set to zero, and the damping coefficient only multiplies the measured velocity. In this way, the damping term is related to the absolute velocity instead of the error velocity, and the controller always provides resistance to the motion, regardless if the user is correctly following or not the desired trajectory (Kim and Deshpande, 2017). In time domain, the control law becomes:

$$\tau =K_{s}\left(\theta_{ref}-\theta_{m}\right)+K_{d}\left({\dot{\theta }}_{m}\right)+\tau_{ref}$$ (5)

Finally, impedance control of 2^*nd*^ order allows to shape also the desired mass/inertia *K*_*i*_ of the system. When dealing with rehabilitation robots, usually the desired mechanical inertia is the one of the human arm, thus the second order term can be neglected. The control law becomes as in Eq. 6, which corresponds to a proportional-integral-derivative (PID) velocity controller.

$$I\left(s\right)=K_{s}+sK_{d}+s^{2}K_{i}$$ (6)

Higher order implementations are possible, and the desired impedance can be set to be of arbitrary order. However, if higher orders are concerned, the impedance control parameters would become physically meaningless, and the computation of high order derivatives can introduce noise to the acceleration signals. In this view, first order impedance control is usually preferred.

**FIGURE 4:**
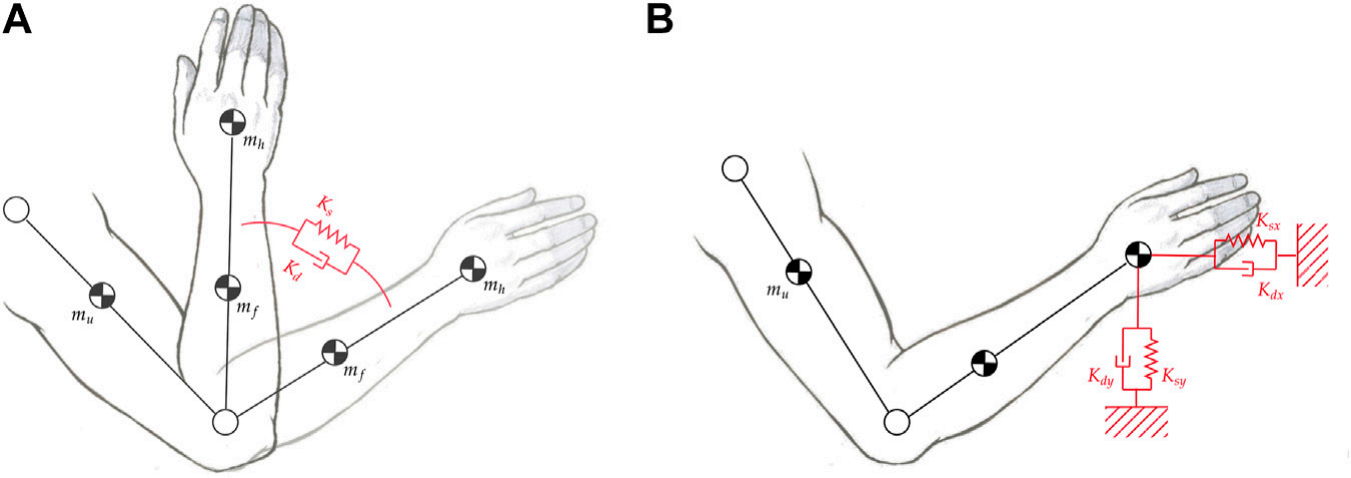
**(A)** First order impedance model applied at the elbow joint in the joint-space. **(B)** First order impedance model applied at the elbow joint in the Cartesian-space.

**FIGURE 5:**
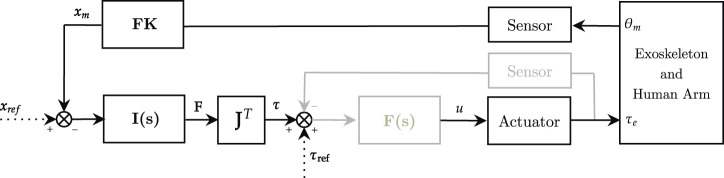
Impedance control scheme in the Cartesian-space. Implicit (black) and explicit (black and gray). **I**(*s*) is the impedance controller, **F**(*s*) is the force controller (only explicit). **FK** represents the forward kinematics model of the exoskeleton, and **J** ^*T*^ corresponds to the transposed Jacobian matrix. *θ*_*ref*_ and *τ*_*ref*_ represent respectively reference angular position and torque, while *u* refers to the motor current control signal.

In rehabilitation robotics, many exoskeletons are controlled in the task-space through Cartesian-space impedance controllers (Frisoli et al., 2009; Nef et al., 2009). This approach is preferred over joint-space impedance control since it favors functional tasks, and it does not require inverse-kinematics algorithms during trajectory generation. The Cartesian-space impedance control scheme is implemented by virtualizing a mechanical impedance in the task-space instead of at the joint level, as shown in Figure 4. While in joint-space the spring-damper system is a rotational system *n*-dimensional (*n* represents the number of active degrees-of-freedom of the robot), in Cartesian-space, the mechanical impedance is linear and three-dimensional. In fact, the corrective action is provided by three-dimensional forces, usually referred to as corrective force-fields. Consequently, in order to permit the robot to generate such assistance, there is the need to convert 3-dimensional task-space forces to *n*-dimensional joint-space torques. Generally, the transposed Jacobian matrix is exploited to compute such conversion. The Cartesian-space impedance control scheme is considered a centralized control approach, since it exploits the robot configuration (usually through forward kinematics) to compute the desired torques at each joint, as shown in Figure 5.

In detail, considering a 1^*st*^ order mechanical impedance on the *x* Cartesian-direction, the force-fields are computed as:

$$F_{x}=K_{s}\left(x_{des}-x_{m}\right)-K_{d}\left({\dot{x}}_{des}-{\dot{x}}_{m}\right)$$ (7)

where *K*_*s*_ and *K*_*d*_ are the desired linear spring and damper, respectively, and *x*_*m*_ is the measured position of the end-effector, computed through the forward kinematics model of the exoskeleton.

In neurorehabilitation, Cartesian-space impedance controllers can be used to implement tunneling strategies. They permit to discriminate robot assistance along the tangential and orthogonal directions of the end-effector reference motion. Thus, the robot can assist along the axial tunnel direction and correct along the radial direction. Cartesian impedance strategies also intrinsically allow time-independent relationships among the exoskeleton joints, which is crucial to enable the user to exhibit voluntary movements. In fact, besides the robot configuration, the ultimate goal is to control the pose of the user’s hand through spring-damper behavior to follow the desired path. However, this strategy (i.e., Cartesian impedance control) does not control or correct compensatory movements or non-coordination among joints. Therefore, it is more prone to maladaptive plasticity mechanisms. For example, Zhang et al. (2020) presented a novel assisted-as-needed controller that, through a task-space impedance controller, assists the position of the hand of the user to follow a virtual tunnel. Stiffness fields are created to push the end-effector to the center of the tunnel and guide it along the tunnel if the user is not fast enough. Furthermore, the proposed controller can be adjusted through five adjustable parameters to implement different robot-aided rehabilitation training such as passive, active-assistive, active, and resistive training.

### 3.2 Admittance Control

**FIGURE 6:**
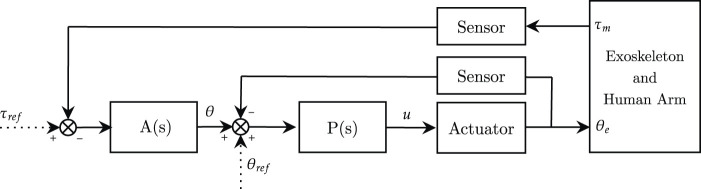
Admittance control scheme in the joint-space. **A**(*s*) is the admittance controller, **P**(*s*) is the position controller. *θ*_*ref*_ and *τ*_*ref*_ represent respectively reference angular position and torque, while *u* refers to the motor current control signal.

Admittance control is the dual approach to impedance control, and it is generally used as a method to promote physical human-robot interaction with stiff, non-backdrivable actuators (Keemink et al., 2018). By definition, admittance control actuates motion (usually position or velocity) through a force/torque feedback, and it is also generally known as position-based impedance control or impedance control with force feedback (Ott et al., 2010). The control scheme involves a nested loop architecture, where the inner loop controls the position (or velocity) of the joint, and the outer torque loop computes the motion setpoint according to the desired human-robot interaction, as shown in Figure 6.

In admittance control, the inner loop “stiffens” the joint, and the outer loop “softens” the human-robot interaction behavior (Calanca et al., 2016). Generally, robot weight compensation is not needed since the robot is position controlled. However, in some works, weight compensation is provided in feedforward at the inner position control loop (**P**(*s*)) to improve trajectory tracking Bai et al. (2017). The main advantage of using admittance control in rehabilitation robotics is that it does not require intrinsic back-drivability of the actuation unit: the inner motion control loop intrinsically compensates and rejects stiction and dynamic friction. In other words, the outer force loop computes the reference motion that produces a virtual backdrivability of the joint (Calanca et al., 2016). When dealing with impedance control, achieving high-fidelity torque control is critical to render a wide variety of mechanical impedance (Z-width), i.e., impedance control requires both high-stiffness gains for good trajectory tracking and low-stiffness gains to promote compliant behavior and its accuracy depends on the capability of the system to deliver high-quality torques. Contrarily, admittance control can exploit the standard features of industrial robots for the implementation of the inner motion loop that can suppress undesired disturbances such as system dynamics and friction, without the need for model-based compensation (Schumacher et al., 2019). The main limitation arises when low-impedance behavior is desired, and high-admittance gains could lead to instability issues. Similar to impedance control, different orders of the admittance filter can be selected. Still, the computation of the reference motion profile in the time-domain may require numerical integration to solve the motion differential equations. In this review, we will consider the admittance model as the relationship between force and position (Ott et al., 2010; Schumacher et al., 2019). In other studies, such as in Calanca et al. (2016), authors described the admittance model as a force-velocity relationship. The admittance model of zero-order is usually referred to as compliance control, and it is formally complementary to stiffness control, by means of the inverse of the desired stiffness *K*_*d*_. The desired zero-order admittance is computed as:

$$A\left(s\right)=1/K_{d}=K_{d}^{-1}$$ (8)

The 1^*st*^ order admittance or accommodation control is one of the most common implementations in rehabilitation since it is suitable for slow motion (Keemink et al., 2018). The motion is derived from the force/torque feedback as follows:

$$A\left(s\right)=1/\left(K_{d}+sD_{d}\right)={\left(K_{d}+sD_{d}\right)}^{-1}$$ (9)

where *D*_*d*_ represents the desired impedance damping (or viscous friction).

For example, Zhuang et al. (2019) proposed a first-order admittance model, characterized by a virtual spring-damper interaction system, to control an ankle rehabilitation exoskeleton promoting compliant behavior. By neglecting the zero-order desired stiffness *K*_*d*_, the impedance model becomes a pure anti-damping velocity-driven admittance controller, which is generally the simplest way to promote transparent behavior at the joint level.

Finally, the second-order admittance model also permits to shape the desired mass/inertia *M*_*d*_ of the virtualized dynamic system. The admittance equation is shown in (Eq. 10).

$$A\left(s\right)=\frac{1}{\left(K_{d}+sD_{d}+s^{2}M_{d}\right)}={\left(K_{d}+sD_{d}+s^{2}M_{d}\right)}^{-1}$$ (10)

The desired stiffness can be removed, and the controller becomes a mass-damper virtualized system, as in Chia et al. (2020).

**FIGURE 7:**
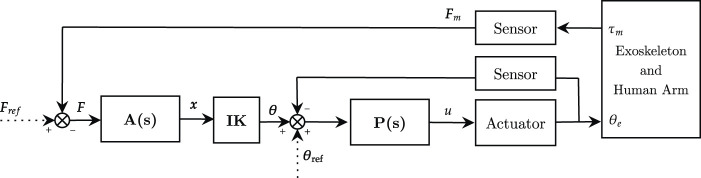
Admittance control scheme in the task-space. **A**(*s*) is the task-space admittance controller, **P**(*s*) is the joint-space position controller. **IK** corresponds to the inverse-kinematics algorithm. *θ*_*ref*_ and *τ*_*ref*_ represent respectively reference angular position and torque, while *u* refers to the motor current control signal.

When coming to rehabilitation exercises, the position-controlled trajectories are generally computed as the sum of the desired joint profiles, namely *θ*_*ref*_, and the angle *θ* that is in turn obtained from the admittance model and the interaction forces. In this way, the robot follows the desired movement but permits deviation according to the user’s voluntary activity. The reference torque *τ*_*ref*_ is normally used to filter out gravity effects and static disturbances from the torque/force measurements *τ*_*m*_, but it can also be tuned to include additional external force-fields to the desired physical human-robot interaction. For upper-limb robots, admittance control in the joint-space (i.e., with torque feedback at the joints level) has not been explored yet, since it requires precise mathematical models for gravity compensation. Instead, 6-DOFs force/torque sensors at the interaction ports of the robot are more often exploited to detect the interaction effort with the user. With this approach, there is no need for gravity compensation of the robot (which is position-controlled), and the force feedback does not need additional filtering for gravity effects. Of course, since the force/torque sensors usually detect interaction in the three-dimensional space, a conversion to joint-space motion is needed to feed the inner control loops (that operate at each joint of the robot). If task-space sensors are used, the conversion can be implemented both for the desired position or for the measured feedback. The admittance control scheme for task-space sensors is shown in Figure 7.

With respect to the task/joint space conversion, Wu et al. (2018b) developed a patient-active admittance controlled exoskeleton for upper-limb neurorehabilitation. Interaction forces with the user are acquired with force/torque sensors at the end-effector. The differential desired forces are then fed to a second-order admittance filter that computes the desired differential trajectory in task space. Finally, an inverse kinematics algorithm computes the desired trajectories in the joint-space. Alternatively, Bai et al. (2017) implemented a second-order admittance control scheme with an upper-limb exoskeleton. The forces are measured at the arm cuffs through force-sensing contact sensors. The sensors measure the human-robot interaction forces, which are analyzed by a “force model controller” that computes the desired interaction torque for each joint. Finally, the admittance filter is applied to compute the motion profiles in the joint-space.

As previously mentioned, in admittance control, the intrinsic back-drivability of the exoskeleton joint is not needed. In fact, the robot is position-controlled by the inner loop, which does not guarantee to be intrinsically transparent to user effort, and external signals are used to detect the intention of movement towards a certain direction. Many different approaches are available to detect user-driven movements. The most common approach relies on direct measurement of interaction effort through force/torque sensors at the interface ports of the robot that are usually at the upper arm and forearm cuffs (Kim and Deshpande, 2017; Wu et al., 2018a; Wu et al., 2018b) (Section 4.2). Alternatively, human voluntary effort can be estimated by means of EMG-based sensing (Zhuang et al., 2019), or with force-sensing resistors (FSR) (Bai et al., 2017).

## 4 Hardware-level Implementation of Compliant Control

When dealing with rehabilitation exoskeletons, most platforms rely on electric motors provided with high-ratio gearboxes to increase the ability to deliver motor torque. However, they are inherently inefficient and they introduce non-linear stiction, static and viscous friction, and reflected inertia, which can compromise back-drivability (Schumacher et al., 2019). Consequently, in most cases the perceived compliance cannot be guaranteed by the mechanical back-drivability of the geared drive itself, and users would need to overcome large torques to initiate voluntary movements (Nef and Lum, 2009). In this review, we focus on electrically powered exoskeletons and we describe three global approaches to promote compliant behavior with geared drives, i.e., to permit the user exert torque onto the robot joint, according to the desired low-level control strategies.

### 4.1 Model-Based Compensation

When dealing with implicit impedance controlled exoskeletons, by which the robot is not provided with additional torque sensors, residual frictional torques need to be compensated by software. A common practice to improve back-drivability of high-ratio gearboxes is to implement friction compensation models (Nef and Lum, 2009; Weiss et al., 2012). In this way, a zero-torque controller could achieve good transparency with low residual resistive torques. Usually, friction is modeled with a kinetic friction term (Coulomb and viscous velocity-dependent) and a breakway friction term, which relates to the stiction phenomena (Armstrong, 1988). The friction compensation is mainly regarded as positive velocity-based feedforward control (Just et al., 2016). Still, the breakaway friction usually cannot be compensated for since the sign of the compensation term depends on the direction of the desired movement, which is not always defined a priori (Nef and Lum, 2009).

### 4.2 Interaction Force/Torque Sensing

Researchers are recently providing their rehabilitation robots and exoskeletons with torque/force sensors, which directly measure the interaction force between the human and the robot (Villani and De Schutter, 2008), namely achieving active compliant control. For impedance control approaches, direct torque sensing permits to implement torque control loops with explicit feedback to reject friction disturbances and to reduce residual resistive torques (Boaventura et al., 2012, 2013). Overall, this approach leads to better torque-tracking performances and improves back-drivability. However, Focchi et al. (2016) demonstrated that high-gain tuning of the torque closed-loop can jeopardize the stability of the robot when touching hard surfaces. Thus, there exists a compromise between low undesired interaction forces and control robustness (Vallery et al., 2008). Torque sensing can also be fed in at the outer loop, such as in admittance control. In this case, the robot is not compliant because of the inner loop, but the effort sensing is used to update the desired trajectory of the inner loop. While torque sensing in impedance control is usually performed in the joint-space, with admittance control, the loadcell is usually installed at the end-effector, through a handle (Ghonasgi et al., 2021), or at the interaction ports, through the arm cuffs that are usually at the upper arm and at the forearm levels (Kim and Deshpande, 2017; Wu et al., 2018a; Kumar et al., 2019). While in most cases the interaction forces are measured with loadcell-based sensors, sometimes force sensing resistors (FSR) are installed inside arm cuffs (Bai et al., 2017).

### 4.3 Mechanical Compliance (Series Elastic Actuators)

While the first two approaches are usually implemented with rigid joints, the perceived compliance can also be implemented by adding mechanical compliance, for example by using soft joints. In fact, compliant control can be also achieved by voluntarily introducing elastic elements (i.e., springs) in series to general purpose electric actuators, namely series elastic actuators (SEA) (Vallery et al., 2008). Several research groups that develop upper-limb exoskeletons are now relying on SEAs (Crea et al., 2016; Chen et al., 2019; Wu et al., 2019) since they are inherently safe, they permit robust force control and they are efficient in periodic tasks (Calanca et al., 2016). Also, if additional mechanical compliance is added to the actuation chain, there is no need for a intrinsic backdrivable geared actuator. However, SEAs, due to the mechanical compliance, result in limited force and position control bandwidth, and can lead to instability issues when trying to achieve high-impedance behavior. Namely, the achievable displayed stiffness of the joint cannot be higher than the physical spring stiffness of the SEA, if passivity is desired (Vallery et al., 2008; Calanca et al., 2017).

The mechanical compliance can potentially simplify the control strategy of the exoskeleton. For example, in Wu et al. (2019); Trigili et al. (2019), compliant control was achieved by position-controlling a SEAs unit. Indeed, there is no need to strictly apply compliant control strategies, since the compliance is intrinsically provided by the physical stiffness. Nevertheless, in most cases, a combination of impedance/admittance control of SEA is used to promote variable impedance behavior (Calanca et al., 2016). In some other cases, if the spring stiffness is high enough, the SEA does not provide a perceivable mechanical compliance, but the spring is used only to indirectly compute the joint torque output by measuring its displacement and to dampen high frequency oscillations (Kim and Deshpande, 2017).

Overall, if impedance control strategies are desired, there is the need for good back-drivability of the joint to promote compliant interaction control. Instead, admittance control typically does not require back-drivable joints, and high transmission ratios are preferred to achieve precise position control, but force/torque sensing is mandatory to detect the intention of movement of the user. Finally, if SEA-based joint are designed, compliance is intrinsically promoted, and torque sensing can be achieved with indirect measurements based on the elastic element deformation.

## 5 Available Exoskeleton Prototypes

This section presents and compares some control solutions for arm rehabilitation exoskeleton prototypes available in the literature. The presented list is non-comprehensive, but works were selected to describe and demonstrate how different approaches could promote similar high-level rehabilitation modalities. Indeed, we focused on works that describe how the three levels of implementation have been combined to obtain a given high-level training behavior. For each solution, we explain its functioning at the three levels of the proposed classification, and we report the key findings of each approach.

### 5.1 The Aalborg University Exoskeleton

The exoskeleton described in Bai et al. (2017); Christensen and Bai (2018) is an upper-limb device provided with three actuated DOFs and one passive DoF, developed at the Aalborg University (AAU). The proposed solution addresses the problem related to the design of the shoulder mechanism, allowing the exoskeleton to match the complex motion of the human shoulder joint. The proposed kinematics relies on a spherical mechanism consisting of two revolute joints connected through a double parallelogram linkage. The exoskeleton is equipped with force sensing resistors (FSR) sensors (capable of detecting physical pressure) to measure the interaction between the user and the exoskeleton. Such measurements are elaborated by a *force model controller* module capable of detecting the direction of the applied interaction forces. Based on the magnitude and direction of the applied forces, the admittance controller has been implemented to provide the subject with the capabilities to operate the exoskeleton based on the applied interaction. Such admittance controller defines the reference velocity for the inner PI trajectory tracking controller with gravity compensation capabilities, allowing to operate the exoskeleton. Preliminary experimental results have shown the performance of the proposed design.

### 5.2 The ALEx Exoskeleton

The Arm Light Exoskeleton (ALEx) is a bimanual robotic device specifically designed for robot-supported rehabilitation of stroke patients. ALEx is a cable-driven, mechanically compliant exoskeleton operated by four actuated and sensorized DOFs (shoulder and elbow) and two passive DOFs (wrist). The robot is equipped with brushless motors located remotely to the exoskeleton joints. The use of compliant cable-based transmission reduces weight and inertia of the system, and the mechanical compliance, introduced by cables, permits to achieve robust force control (Stroppa et al., 2017a). In its first version, ALEx allows to perform movements in three different modalities: 1) *robot-passive*, namely human-active modality according to our classification (i.e., the participant moves the arm in the workspace and the robot follows the motion), 2) *robot-active*, namely human-passive modality according to our classification (i.e., the robot guides the participant’s arm during the movement), and 3) *assisted-as-needed*, or passive-triggered modality, by which the robot guides the motion only if a timeout-based trigger is reached (Pirondini et al., 2016). At the low-level, the exoskeleton can be operated in *force mode*, which provides desired forces at the end-point, or in *compliant position mode*, which relies on independent position control of the robot compliant joints. For all modalities, the low-level force controller is computed as the sum of several feedforward contributions, including gravity compensation of the moving robotics links, friction compensation of transmission mechanisms, and inertia compensation of moving parts. In a recent study, Stroppa et al. (2017b) presented an adaptive assistance controller based on a Cartesian-space impedance control scheme. The impedance law is based on a mass-spring-damper dynamic system that corrects joint trajectories in the task-space. Finally, the assist-as-needed paradigm is implemented according to an online performance evaluation of the subject’s motor skills.

### 5.3 The ARAMIS Exoskeleton

The ARAMIS exoskeleton is a bi-manual exoskeleton for upper-limb neurorehabilitation after stroke. The robotic platform includes two fully motorized 6-DOFs symmetric exoskeletons (Pignolo et al., 2012). The robot can operate in different modalities that involve the use of both the unaffected and the paretic arm (Pignolo et al., 2016). In *synchronous mode*, which can be addressed as master-replica mode, the robot supports the impaired limb of the subject and replicates the sample movements performed by the other arm in real-time. In *asynchronous mode*, instead, the contralateral exoskeleton arm first records a sample movement, then the robot actively supports the paretic arm along the mirrored task. Such trajectories can be recorded either by the patient’s unaffected limb or by the therapist’s guidance (Dolce et al., 2009). The ARAMIS exoskeleton can also operate in *weight counterbalance mode*: the robot compensates for the arm weight during movements replicating those executed by the contralateral side (Pignolo et al., 2016). Each joint of the robot is actuated by DC brushed motors coupled with high transmission ratio gearboxes. To enhance the backdrivability of the system, the authors developed an integrated joint that relies on a SEA-based design (Colizzi et al., 2009). Series springs are connected at the output shaft of the actuator, and a secondary encoder measures the spring displacement. In this way, the controller can detect whether the patient initiates the movement and the exoskeleton follows the user-driven action.

### 5.4 The ARMin exoskeleton(s)

The ARMin exoskeleton is an upper-limb rehabilitation robot developed at the Sensory-Motor Systems Lab, ETH Zurich. The exoskeletal system has been conceived to promote task-oriented, repetitive, and intensive arm training in patients with upper extremities paralysis (Nef et al., 2009). In its early versions, ARMin is actuated with six DOFs. Four of them are controlled by implicit impedance control laws, while two are operated in admittance mode (Oldewurtel et al., 2007). The two DOFs controlled in admittance promote shoulder elevation and translation movements and rely on force/torque measurement of a 6-axis sensor at the shoulder joint. The robot is embedded with model-based friction and inverse dynamics compensation to improve transparency and minimize interaction forces with the human limb. In Nef et al. (2007), the ARMin II can operate in two different high-level modalities, according to the patients’ recovery stage. In *passive mobilization mode*, first, the therapist moves the patient’s arm together with the robot on the desired trajectory. In this phase, the robot’s gravity and friction are compensated so that the therapist feels only the forces and torques necessary to move the human arm. Then, an algorithm extracts relevant way-points during the movement and computes a minimum jerk trajectory to be followed by the passive mobilization therapy with adjustable velocity. The authors also implemented a ball game based on an *assistive mode*: the subject has to catch a virtual ball rolling down an inclined virtual table. Through a Cartesian-space impedance control law, the robot supports the user by driving their hand along the horizontal plane with gradient force-fields assistance that pushes the patient’s arm towards the ball position. Guidali et al. (2011) presented a further development of ARMin III. The authors built a virtual tunnel around the task-space reference trajectory allowing the user to move freely within the tunnel while guided at the tunnel walls. Furthermore, the exercise could be triggered by the patients’ voluntary activity when the user effort overcomes a certain threshold, and the movement is directed to the next task. In a recent study, the researchers improved the ARMin IV exoskeleton’s transparency through a velocity-based disturbance observer. They compared it to the more traditional friction, gravity, and inertia feedforward compensation (Just et al., 2018). Finally, three distinct methods for arm weight compensation using the ARMin exoskeleton were proposed and analyzed (Just et al., 2020). All three methods are based on anthropometric arm models and are generalizable for use in different robotic devices and various subjects.

### 5.5 The CleverARM Exoskeleton

The CLEVERarm is an 8-DOFs lightweight and ergonomic upper-limb rehabilitation exoskeleton for upper-limb impairment developed at the Texas A&M University, capable of producing diverse and perceptually rich training scenarios Soltani-Zarrin et al. (2017b); Zeiaee et al. (2019). The robot supports the motion of the shoulder girdle, glenohumeral joint, elbow, and wrist. Six degrees of freedom of the exoskeleton are active, and the two degrees of freedom supporting the wrist motion are passive. The CLEVERarm joints employ electric motors coupled with harmonic drive actuators, and the mechatronic system is provided with force/torque sensors at the two interaction points with the arm to enable achieving back-drivability of the exoskeleton. The control scheme relies on an impedance-based controller employed to track rehabilitation exercises implemented in the game environment. The *path generator* computes human-like motions that support the scapulohumeral rhythm Soltani-Zarrin et al. (2017). Additionally, the controller is provided with a gravitational model of the robot to cancel the weight of the exoskeleton, and a friction compensation method, achieved through an admittance-based controller. Finally, the interaction forces measured by the F/T sensors are used to compute the desired velocity of the interaction ports to improve the back-drivability of the exoskeleton. Then, the desired angular velocities are computed through the system Jacobian and are fed as references to the impedance-based controller Soltani-Zarrin et al. (2018).

### 5.6 The EXO-UL8 Exoskeleton

The EXO-UL8 is a dual-arm exoskeleton that covers all the main movements of a human’s upper limb. The robot supports the motion of the shoulder, elbow, and wrist through seven non-backdrivable joints, and an additional joint operates the handgrip Shen et al. (2019). In a previous version, the EXO-UL7 was actuated through cable-driven actuation mechanisms. Now, the EXO-UL8 is operated by electric motors coupled with harmonic drives. A set of four force/torque sensors are placed at the physical interaction points between the user and the exoskeletal system. The robot relies on an admittance controller that allows the exoskeleton to behave transparently to user-driven movements. Precisely, torques applied by the human to the exoskeleton joints are estimated from the F/T sensors, then, through an admittance model, reference trajectories are generated and operated by the inner low-level PID control loops. Friction and gravity compensation is added as feedforward terms to the low-level controller Shen et al. (2019). The core concept of the EXO-UL8 controller is to generate motion in response to human-applied forces to improve backdrivability and reduce the user-perceived weight of the robot. On top of this, the system can be operated to follow pre-defined trajectories for rehabilitation exercises. Since the authors developed a dual-arm symmetric system, they also enabled mirroring training modes based on bilateral teleoperation between unimpaired and impaired arms. Additionally, Shen et al. (2018) proposed an asymmetric bilateral training using an interactive virtual reality environment.

### 5.7 The FELXO-Arm1 Exoskeleton

The FELXO-Arm1 system is an upper-limb exoskeleton for neurorehabilitation. It supports shoulder and elbow movements through 5-DOFs aligned with human upper-limb joints to match natural physiological synergies. Lin et al. (2021) presented a control strategy for customized robot-assisted passive rehabilitation. The method aims to coordinate shoulder and elbow movements during the early stage of the rehabilitation treatment. The authors proposed a teaching training strategy by which the therapist provides desired trajectories by driving the impaired limb of the patient in the workspace. The trajectory is then adjusted in position, velocity, and acceleration to promote movement smoothness and continuity. Then, the movement is repeated over time with high intensity. The exoskeleton joints are controlled by a proportional differential-based trajectory tracking controller based on an implicit impedance control law. The inverse dynamic model of the system is computed according to the Lagrange method, and the generated torques are included as a feedforward torque controller. The FELXO-Arm1 exoskeleton is powered by BLDC electric motors coupled with harmonic drive gearboxes. A friction compensation algorithm rejects residual dynamic and breakthrough friction at each joint to improve transparency and compliant behavior. Torque sensors measure human-robot interactive torques at shoulder and elbow joints to identify the movement intention of the patient.

### 5.8 The Harmony Exoskeleton

Kim and Deshpande (2017) developed an upper-body bi-manual exoskeleton for post-stroke rehabilitation aimed at providing natural coordination at the shoulder complex. The robotic exoskeleton consists of seven degrees-of-freedom (DOFs) for each arm: five DOFs are used to assist the shoulder and the scapulohumeral rhythm, one DOFs assists the elbow flexion/extension, and one operates the wrist pronation/supination. The authors developed a baseline controller that implements active modalities with joint-coordination constraints (Kim and Deshpande, 2015; Dalla Gasperina et al., 2020). The *baseline controller* promotes joint transparency, corrects for non-coordinated scapulohumeral rhythm through an impedance control law (spring-damper corrective assistance), and compensates for the robot weight through positive feedback that is computed inverse dynamics recursive algorithm (weight counterbalance assistance). The exoskeleton is actuated with SEAs, which are used to compute an indirect measure of torque by measuring the deformation of the elastic element. The exoskeleton can also be operated with an explicit joint-space impedance control scheme to follow desired trajectories Oliveira et al. (2019), and it can promote different high-level modalities ranging from *assistive* to *resistive* modalities. Finally, the mechatronic system is also provided with force/torque 6-axis sensors at the interaction upper arm and forearm cuffs, and it can be operated to display a desired stiffness in the Cartesian space as well (Kim and Deshpande, 2017).

### 5.9 The L-EXOS Exoskeleton

Frisoli et al. (2005) developed a force-feedback cable-driven light exoskeleton (L-EXOS) for rehabilitation. The L-EXOS robot operates four active DOFs: three DOFs assist the shoulder ball-socket joint, and one DOF is devoted to the elbow movements. In Frisoli et al. (2009), the robot is controlled in *assistive mode*, and the robot assists the motion only when the subject is not able to complete the rehabilitation exercise. In detail, the controller discriminates longitudinal and orthogonal direction with respect to the reference trajectory and promotes a virtual tunnel that follows the task-space desired trajectory. From a lower-level perspective, two concurrent task-space impedance controllers act along the tangential and orthogonal directions of the trajectory and compute the desired restoring forces at the end-effector. The transposed Jacobian matrix is used to convert task-space forces to joint-space torques, that are actuated accordingly. The dynamic model of the exoskeleton is derived from CAD models and the weight compensation of the device is implemented with a feedforward torque contribution. The robot is actuated with permanent magnet torque actuators, that do provide intrinsic backdrivability. Also, the design of the exoskeleton was conducted following a set of guidelines to improve the transparency of the device, such as choosing high power density actuators, low transmission ratios, and low backlash gearboxes. Furthermore, motors were placed remotely with respect to the actuation joint using tendon transmissions to minimize the perceived inertia due to the motors’ weight.

### 5.10 The NEUROExos Shoulder-Elbow Module Exoskeleton

The NEUROExos Shoulder-Elbow Module (NESM) is an exoskeleton for upper-limb neurorehabilitation and spasticity treatment. It actuates 4-DOFs, namely 3-DOFs at the shoulder and 1-DOF at the elbow (Crea et al., 2016). The exoskeleton joints are composed of high-torque SEA units, which permit high-fidelity torque control and introduce mechanical compliance to accommodate users’ voluntary movements. Torque-sensing is achieved in joint-space by indirect measurement on the series-spring displacement. The robot can operate in various training modalities, such as passive mobilization, active-assisted, active-resisted, and active-disturbed training modes. In order to adapt the robot assistance to a wide range of patients’ residual movements, the exoskeleton is provided with two control macro-modalities: *robot-in-charge* and *patient-in-charge* programs (Trigili et al., 2019). In the *robot-in-charge* approach, the robot passively mobilizes the human arm along pre-defined joint trajectories. The joints are position-controlled through a standard PID scheme, and the intrinsic serial elasticity provides additional compliance to accommodate spasticity and uncomfortable positions. Join trajectories are computed through an inverse-kinematics algorithm by selecting maximum joint or hand velocities. Instead, in *patient-in-charge* mode, each joint of the exoskeleton is torque-controlled. In particular, the feedback torque is derived from the series-spring elongation, and a PID scheme tracks the desired torque. In this macro-mode, the robot promotes compliant control, behaves transparently to user-initiated movements, assists and resists the user’s movements at each joint according to the desired training modality. The authors also present some additional sub-modes. In transparent mode, the robot tracks the null-torque and compensates for its weight. In impedance control mode, a dual (convergent and divergent) explicit impedance control scheme assists (or disturbs) the motion along the desired joint trajectories. Finally, three variants of muscle strength training modes are implemented to training specific muscular groups in a resistive-like manner. In all patient-in-charge sub-modalities, a gravity compensation algorithm iteratively computes the gravity torque of each joint due to the robot weight. Then, the gravity torque is fed as a torque feedforward contribution to the central controller (Crea et al., 2017).

### 5.11 The NTUH-II Exoskeleton

The NTUH-II exoskeleton is an upper-limb device for robotic rehabilitation for shoulder-impaired patients (Lin et al., 2014). Such exoskeleton is provided with 8 DOFs, being able to reproduce most of the shoulder movements, such as shoulder flexion/extension, horizontal abduction/adduction, and rotation. The exoskeleton has been provided with the following control schema (Chia et al., 2020). A Kalman filter has been designed in order to estimate the human torques. An admittance model is then used to access the active motion of the human (therefore, making it possible to estimate the user’s intention of motion). On top of that, a velocity field is designed in order to provide active assistance to the subject in interaction with the exoskeleton. Finally, an integration method is proposed in order to combine the admittance model output with the velocity field output, providing the reference signal to the exoskeleton controller, computing the torques to be applied by the motors. The main contribution that has been given by the proposed control approach is related to the velocity field: it provides a method for the generation of time-independent assistance based on the given rehabilitation task. In order to implement the proposed velocity field, the considered rehabilitation task has to be parameterized. After that, the path is encoded using the velocity field to make the assistance time-independent. The adopted velocity field is capable to assist the subject to execute the target task along the tangential direction of the reference trajectory while compensating for deviations along the normal directions. Experimental results have demonstrated the capabilities of the proposed approach to assist the subject during the rehabilitation task execution.

### 5.12 The Pneu-WREX Exoskeleton

The Pneu-WREX exoskeleton is an upper-limb device for robot-aided movement training following stroke (Wolbrecht et al., 2008). The Pneu-WREX exoskeleton is provided with 4 pneumatically actuated DOFs. The following three main characteristics have been implemented in the proposed device: mechanical compliance, the ability to assist patients in completing desired movements, and the ability to provide only the minimum necessary assistance. In order to provide active assistance to the subject, the exoskeleton is controlled exploiting two control loops: an inner controller based on a standard model-based, adaptive control approach in order to learn the patient’s abilities and assist in completing movements while remaining compliant, and an outer *assistance-as-needed* controller defining a force term to the adaptive control law. Such an outer controller decays the force output from the robot when errors in task execution are small, while it increases the assistance to the user when errors in task execution are bigger. The proposed controller has been demonstrated to be successful in experimental tests executed with people who have suffered a stroke.

### 5.13 The RECUPERA Exoskeleton

Kumar et al. (2019) recently presented a lightweight dual-arm rehabilitation robot called RECUPERA exoskeleton. The exoskeleton offers a high level of modularity. It can be used as a wheelchair-mounted system or as a full-body system for therapist-guided and self-training for neurorehabilitation of the upper body. The wheelchair-mounted version features 5-DOFs for each arm, while the full-body operates 30 DOFs. The RECUPERA exoskeleton implements three different rehabilitation training modalities, namely *gravity compensation*, *teach-and-replay*, and *master-slave* therapy. In *gravity compensation* mode, the robot compensates for its weight through an inverse dynamic model of the exoskeleton. This mode can also include the compensation of the human arms dynamic model, and it is conceived as the baseline controller of the robot. The *teach-and-replay* mode consists of two phases. First, the robot is operated in gravity compensation mode, and the therapist performs a trajectory that is recorded by the system. Afterward, the robot detects a trigger from the user (or from the therapist) and passively performs the recorded movement. Finally, the *master-slave* mode consists of a mirroring strategy by which the paretic arm follows and mimics the trajectory performed with the healthy arm operated in gravity compensation mode. The RECUPERA exoskeleton is powered by high-torque BLDC actuators coupled with low-backlash gearboxes to increase torque output at the joint axis. The joints are controlled with cascaded position, velocity, and current control loops, while torque control is achieved through motor current measurements. The exoskeleton is also provided with 6-DOFs force/torque sensors to detect human-robot interaction at the three interfaces: hand, forearm, and upper arm interaction ports.

## 6 Discussion and Perspective

Control advancements for upper-limb exoskeletons for rehabilitation are spreading rapidly, and the literature continuously presents new prototypes and control approaches. Since we noticed that state-of-the-art reviews on controls for rehabilitation robots generally focus their attention on training modalities and human-robot interaction, our study was intended to propose a multi-disciplinary taxonomy of patient-cooperative control strategies for rehabilitation upper-limb exoskeletons that could help researchers develop complex and advanced systems. Our classification is based on a three-level scheme: on 1) *high-level training modalities*, 2) *low-level control strategies*, and 3) *hardware-level implementation*. Overall, we report that most high-level modalities are based on assistive approaches, by which the robot partially supports the user during the motion. In turn, most exoskeletons support human movements in three ways. On one side, they provide corrective assistance, either through impedance-based strategies or via tunneling methods that guide the user to stay within a specific virtual path. Alternatively, weight counterbalance, also known as anti-gravity support, can be implemented to compensate for gravity due to the user’s arm. Finally, inter-joint coordination is involved whether the aim is to induce physiological coordination based on position, torque, or velocity-based synergies.

As shown in Table 3, different “low-level” control strategies can be used to promote the same “high-level” modalities, and there is not a unique relationship between the three layers. The majority of upper-limb exoskeletons are conceived upon the foundation of compliant control, by which the user should have the lead when performing the rehabilitation task. The robot should always follow the user’s intention and apply corrective actions only when the residual muscular forces are insufficient to fulfill the action. Among low-level compliant control strategies, impedance control is the most used since it outperforms admittance control strategies when low-impedance behavior (i.e., transparent free motion) is desired. Furthermore, we underline the importance of achieving high-quality compliant control, either through high-fidelity torque control in conjunction with impedance control strategies or through torque/force-sensing combined with admittance control. Indeed, many researchers rely on force feedback, which can be obtained either via SEA-based indirect measurements or torsional/linear loadcell-based direct measurements. Many studies demonstrated that torque feedback could improve the performances of compliant control over model-based compensation methods. However, the introduction of additional sensors can drastically increase prototype costs. Series elastic actuators are gaining increasing interest since they provide inexpensive, accurate torque sensing at the joint and introduce mechanical compliance to promote compliant motion. Furthermore, with SEAs, it is impossible to achieve higher stiffness than the elastic element, reducing the robot performances when high-impedance (rigid) interaction is required.

**TABLE 3:** Comparison of available control strategies at high-level, low-level and hardware-level for upper-limb neurorehabilitation exoskeletons. **S**: shoulder, **E**: elbow, **W**: wrist.

Exoskeleton	Supported joints	DOFs	Bimanual	High-level	Low-level	Actuation	Sensing	References
**AAU**	S,E	3	no	None	Admittance (with gravity compensation)	Brushless motor coupled with harmonic drive gearbox	Task-space force sensing resistors (FSR)	Bai et al. (2017)
**ALEx**	S,E	4 × 2	yes	Passive (passive-triggered), Active-assistive (adaptive assistance) and Active modalities	Position control of compliant joints, Task-space force control, Cartesian impedance control (with friction, gravity and inertia compensation)	Brushless DC motor coupled with mechanically compliant cable transmission	no	Pirondini et al. (2016), Stroppa et al. (2017b)
**ARAMIS**	S,E,W	6 × 2	yes	Passive (mirroring), Active-assistive (counterbalance)	Position control of SEA-based joints	SEA-based brushed DC motor coupled with gearbox	Joint-space (SEA-based indirect)	Colizzi et al. (2009), Pignolo et al. (2012, 2016)
**ARMin**	S,E,W	6	no	Passive, Active-assistive (corrective, tunneling)	Joint-space and task-space implicit impedance control (with friction and dynamics compensation)	Brushed DC motor coupled with harmonic drive gearbox	Task-space (6-axis F/T sensor, only ARMin II and III)	Nef et al. (2009), Guidali et al. (2011), Just et al. (2020)
**CLEVERarm**	S,E,W	6	no	Passive, and Active-assistive	Joint-space impedance control (with friction and gravity compensation) and admittance-based control for back-drivability	Electric DC motor coupled with harmonic drive gearboxes	Task-space (6-axis F/T sensors)	Soltani-Zarrin et al. (2017b)
**EXO-UL8**	S,E,W	7	yes	Passive and Active-assistive (symmetric and asymmetric mirroring), Active (transparent) modalities	Task-space admittance control (with friction and gravity compensation) and inner joint-space position control	Electric DC motor coupled with harmonic drive gearboxes	Task-space (6-axis F/T sensors)	Shen et al. (2018, 2019)
**FELXO-Arm1**	S,E	5	no	Passive (teach-and-replay), Active-assistive	joint-space implicit impedance control	Brushless DC motor coupled with harmonic drive gearboxes	Joint-space (direct)	Lin et al. (2021)
**Harmony**	S,E,W	7 × 2	yes	Active-assistive (corrective and counterbalance), Active (inter-joint coordination) and Resistive modalities	Explicit impedance control (with friction and dynamics compensation)	SEA-based brushless DC motor	Joint-space (SEA-based indirect), task-space (6-axis F/T sensors)	Kim and Deshpande (2017)
**L-Exos**	S,E	4	no	Active-assistive (corrective), Active (tunneling)	Task-space implicit impedance control (with friction and gravity compensation)	Quasi-backdrivable permanent magnet torque motor	no	Frisoli et al. (2005, 2009)
**NESM**	S,E	4	no	Passive, Active-assistive, Resistive (viscous-field and error-augmentation)	Position control of SEA-based joints, joint-space explicit impedance control	SEA-based brushless DC motor coupled with harmonic drive gearboxes and custom springs	Joint-space (SEA-based indirect)	Crea et al. (2016); Trigili et al. (2019)
**NTUH-II**	S,E,W	8	no	Active-assistive (velocity-field control)	Admittance control	Brushless DC motor coupled with gearbox	Task-space (6-axis F/T sensors at wrist and upper arm)	Lin et al. (2014); Chia et al. (2020)
**Pneu-WREX**	S,E	4	no	Active-assistive (assistance-as-needed)	Model-based adaptive impedance control	Pneumatic actuation	no	Wolbrecht et al. (2008)
**RECUPERA**	S,E,W	5 × 2	yes	Passive (mirroring), Active-assistive (counterbalance)	Position, velocity, current control	Brushless DC motor coupled with low-backlash gearboxes	task-space (6-axis F/T sensors)	Kumar et al. (2019)

**FIGURE 8:**
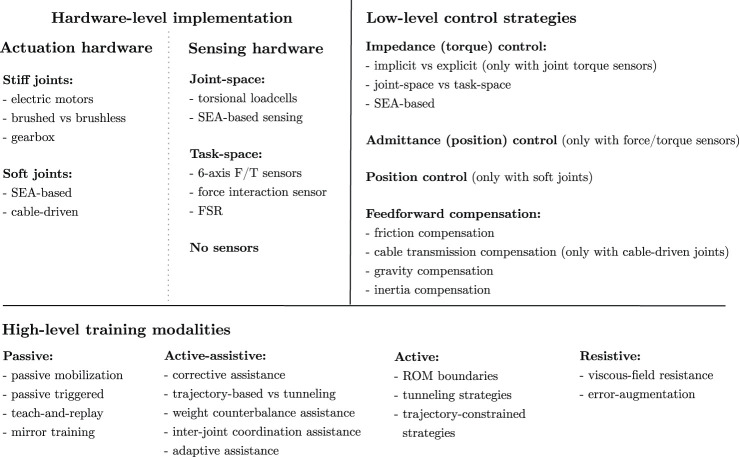
Summary of patient-cooperative control strategies for upper-limb rehabilitation exoskeletons at different levels of implementation: hardware-level actuation and sensing implementation, low-level control strategies, and high-level training modalities.

In Figure 8, we report a summary of the aspects we investigated in this work, intending to help robotics researchers bridge the gap between desired rehabilitation outcomes and robotic implementation. The reader can interpret the scheme following both bottom-up and top-down paradigms: 1) the exoskeleton control design process can both start from the available hardware (actuator, sensors, etc.) to define the implementable control strategies for the desired behavior, or 2) researchers can identify the hardware requirements from the selected high-level training modalities.

In conclusion, this review presents an interdisciplinary vision on control solutions for upper-limb exoskeletons that suggest how different approaches can render physical human-robot interaction at different levels of implementation to promote the desired rehabilitation behavior. We noticed that most high-level training modalities are derived from motor learning concepts to improve the rehabilitation outcomes, and exoskeletons are usually programmed to mimic the therapist’s actions during conventional treatment. We suggest that to exploit robotic assistance to promote motor recovery, more neurological-inspired modalities should be investigated to deepen the effects of robot-mediated therapy on neural plasticity and motor relearning. Finally, further research is needed to evaluate which approach (at each level) is associated with a more significant improvement of arm functions after stroke.
